# Expression and characterization of recombinant antibodies against H7 subtype avian influenza virus and their diagnostic potential

**DOI:** 10.3389/fmicb.2024.1459402

**Published:** 2024-08-23

**Authors:** Siwen Wang, Ying Zhang, Xu Zhou, Yue Ma, Jianzhong Shi, Yongping Jiang, Yanbing Li, Guobin Tian, Xiurong Wang

**Affiliations:** ^1^State Key Laboratory for Animal Disease Control and Prevention, Harbin Veterinary Research Institute, Chinese Academy of Agricultural Sciences, Harbin, China; ^2^College of Life Engineering, Shenyang Institute of Technology, Shenyang, China

**Keywords:** avian influenza viruses, H7 subtype, recombinant antibody, monoclonal antibodies, CHO-S

## Abstract

**Introduction:**

Monoclonal antibodies (mAbs) play a pivotal role in disease diagnosis as well as immunotherapy interventions. Traditional monoclonal antibody generation relies on animal immunization procedures predominantly involving mice; however, recent advances in *in-vitro* expression methodologies have enabled large-scale production suitable for both industrial applications as well as scientific investigations.

**Methods:**

In this study, two mAbs against H7 subtype avian influenza viruses (AIV) were sequenced and analyzed, and the DNA sequences encoding heavy chain (HC) and light chain (LC) were obtained and cloned into pCHO-1.0 expression vector. Then, the HC and LC expression plasmids were transfected into CHO-S cells to establish stable cell lines expressing these mAbs using a two-phase selection scheme with different concentrations of methotrexate and puromycin. Recombinant antibodies were purified from the cell culture medium, and their potential applications were evaluated using hemagglutination inhibition (HI), western blotting (WB), confocal microscopy, and enzyme-linked immunosorbent assay (ELISA).

**Results:**

The results indicated that the obtained recombinant antibodies exhibited biological activity similar to that of the parent antibodies derived from ascites and could be used as a replacement for animal-derived mAbs. A kinetic analysis of the two antibodies to the AIV HA protein, conducted using surface plasmon resonance (SPR), showed concordance between the recombinant and parental antibodies.

**Discussion:**

The data presented in this study suggest that the described antibody production protocol could avoid the use of experimental animals and better conform to animal welfare regulations, and provides a basis for further research and development of mAbs-based diagnostic products.

## 1 Introduction

Avian influenza viruses (AIVs) are classified into low-pathogenicity avian influenza (LPAI) and highly pathogenic avian influenza (HPAI) viruses. Most of the HPAI viruses belong to the H5 or H7 types, among them, first reported HPAI in history is the H7 subtype, which can cause severe disease and high mortality in infected poultry, leading to devastating impacts on the poultry industry. In early 2017, outbreaks of H7N9 HPAI on chicken farms in China caused considerable damage to the poultry industry (Shi et al., 2018, 2023). In 2020, an outbreak of H7N7 HPAI on farms in Australia led to a substantial loss of livestock and had a significant economic impact (Bisset and Hoyne, 2021).

Historically, the H7 subtype avian influenza virus has caused human infections multiple times. For example, an outbreak of subtype H7 infections in humans occurred in spring 2003 when an HPAI H7N7 virus was detected on commercial poultry farms in the Netherlands (Munster et al., 2007). Between 2013 and 2017, five waves of H7N9 infection occurred in China, and the number of laboratory-confirmed cases and human deaths were 1,568 and 615 (Munster et al., 2007; Chen et al., 2013; Gao et al., 2013; Kile et al., 2017; Yin et al., 2021), respectively. H7 subtype influenza viruses not only have caused outbreaks in poultry in many countries, resulting in huge losses to the industry, but also have caused serious respiratory diseases in humans, with some even leading to death (Shi et al., 2017; Wan et al., 2022).

Monoclonal antibodies (mAbs) are crucial for the development of diagnostic reagents. Recombinant mAbs, produced through molecular cloning techniques, offer high batch consistency, and repeatability. Recombinant mAbs methods avoid the slight changes in antibody production caused by genetic drift in hybridoma cell lines as well as the use of mouse ascites and therefore the need for animal ethics board review. An additional advantage of recombinant antibodies is their potential for increased versatility, as researchers can genetically fuse the primary amino acid sequence to other molecules, such as fluorophores, to generate custom tools with diverse functionalities.

**Figure 1:**
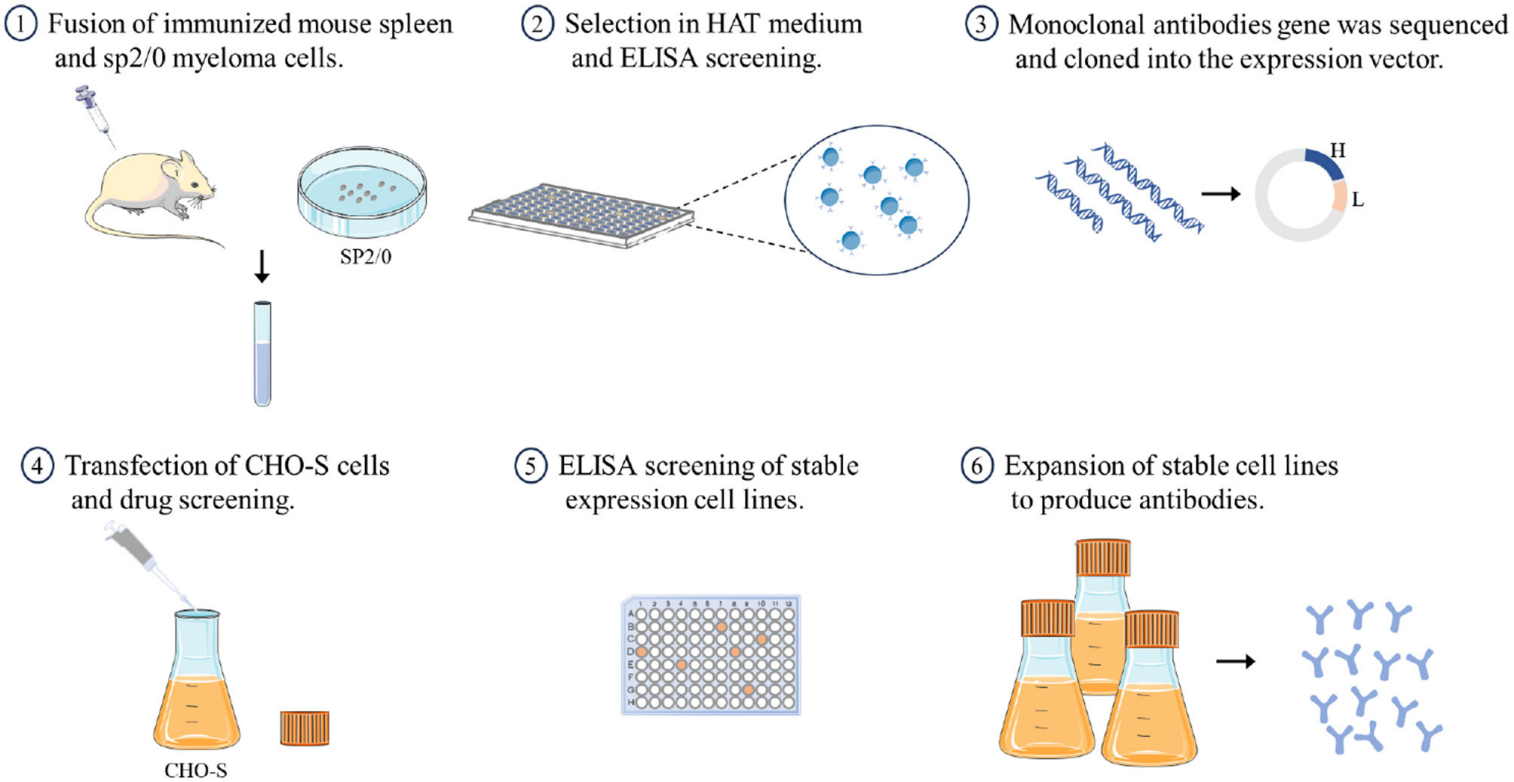
A brief overview of the process for preparing recombinant antibodies.

In this study, we sequenced two mAbs against H7 subtype AIV derived from hybridoma cells and cloned them into an expression vector. We then established two stable cell lines that express these mAbs and obtained recombinant mAbs from the cell culture supernatant (Figure 1). Testing revealed that the biological activities of the recombinant mAbs were similar to those of the parent antibodies, demonstrating that antibodies expressed *in vitro* can effectively replace ascites-derived parent antibodies for use in diagnostics or basic research.

## 2 Materials and methods

### 2.1 Viruses and cells

The A/chicken/Guangdong/SD008/2017(H7N9) virus, abbreviated hereafter as CK/SD008, was isolated and stored at the State Key Laboratory for Animal Disease Control and Prevention as previously described (Shi et al., 2017). SP2/0-AG14 myeloma cells and HEK-293T cells were cultured in DMEM (Gibco) containing 10% fetal bovine serum (ExCell) at 37°C and 5% CO_2_. CHO-S cells were cultured in CD FortiCHO™ Medium (Gibco) supplemented with Anti-Clumping Agent (Gibco) and GlutaMAX™ (Gibco) at 37°C and 8% CO_2_.

### 2.2 mAbs preparation and sequencing

Virus CK/SD008 was proliferated in specific-pathogen-free (SPF) embryonated chicken eggs, inactivated with β-propiolactone, and purified by sucrose density gradient centrifugation. Three 6-week-old female BALB/c mice were immunized with intraperitoneal and subcutaneous injections of purified protein from CK/SD008 emulsified with Freund's adjuvant (Sigma-Aldrich). Splenocytes were then fused with SP2/0-AG14 myeloma cells at a ratio of 10:1 with polyethylene glycol (PEG) (Sigma-Aldrich) according to standard procedures. The fused cells were screened in complete medium containing hypoxanthine-aminopterin-thymidine (HAT) (Sigma-Aldrich). Positive hybridoma cell lines were detected by indirect enzyme-linked immunosorbent assay (ELISA) and hemagglutination inhibition (HI). After three subcloning runs, the optimal single-cell mass was obtained.

Total RNA was extracted from hybridoma cells, and cDNA was synthesized by reverse transcription using M-MLV Reverse Transcriptase (Invitrogen) and oligo (dT) primers. The major variable (V) and partial constant (C) regions of the heavy and light chains were amplified by multiple PCR runs and sequenced using a DNA analyzer (3500XL Genetic Analyzer; Applied Biosystems). The C-region gene fragment with the highest homology was screened against GenBank using Igblast (2023), and primers were designed to amplify the C region using PCR. Polyadenine deoxynucleotides were added to the 3′ end of the cDNA by terminal deoxynucleotide transferase (Thermo Scientific), and the 5′ end of the cDNA was amplified using connector primers and gene-specific primers (5′ RACE), and then the 3′ end of the cDNA was amplified using the 3′ end of the rapid amplification technology (3′ RACE). Finally, the complete DNA sequences of the heavy and light chains of the mAbs were obtained.

### 2.3 Expression and purification of the recombinant mAbs

DNA fragments of the heavy and light chains were cloned into the pCHO-1.0 expression vector and transfected into CHO-S cells via electroporation. The transfected cells were screened in medium containing 10 or 20 μg/mL puromycin (Gibco) for 7 days. Next, methotrexate (100, 200, 300, 400, 500, 600, 700, 800, 900, and 1,000 nM; Sigma-Aldrich) was used for pressure screening, and the cells were treated with the drug at each of the above concentrations for 7 days. The medium was changed every 3 days during screening. Positive cells were inoculated into medium at a concentration of 5 × 10^5^ cells/mL and incubated for 10 days. The glucose concentration was maintained at 5 g/L. Then, the culture supernatant was collected, and the antibody was purified using the AKTA Prime Plus System (GE Healthcare) and a HiTrap Protein G HP antibody purification column (Cytiva).

### 2.4 Hemagglutination inhibition assays

Hemagglutination inhibition (HI) assays were performed according to the WOAH Manual (WOAH, 2023). Briefly, 25 μL of each sample was serially (two-fold) diluted with PBS (pH 7.4) in a V-bottom 96-well microtiter plate. Then, antigen (25 μL of 4 HA units of CK/SD008) was added to the plates and incubated at room temperature. After 30 min, 25 μL of 1% chicken red blood cell (RBC) was added to all wells and incubated at room temperature for 40 min. The reciprocal of the highest dilution of the antibody was defined as the HI titer.

### 2.5 Western blotting

The purified CK/SD008 proteins were treated with RIPA lysate buffer (YEASEN), mixed with 4 × Protein Native PAGE Loading Buffer, separated by polyacrylamide gel electrophoresis (ExpressPlusTM PAGE Gels; GenScript), and then transferred to nitrocellulose (NC) membranes using an eBlot™ L1 (GenScript). The NC membranes were blocked with 5% skim milk at 37°C for 1 h and then incubated with purified mAb that was either expressed in CHO cells or obtained from ascites at 4°C overnight. Then, the membranes were incubated with IRDye^®^ 800CW Goat Anti-Mouse IgG (Li-Cor BioSciences) at room temperature for 1 h, scanned, and analyzed with an Odyssey CLX infrared imaging system (Li-Cor BioSciences).

### 2.6 Confocal microscopy

HEK-293T cells seeded in glass-bottom dishes were transfected with PCAGGS-CK/SD008-HA-flag plasmid (1.5 μg) or the PCAGGS empty vector as a mock/control. At 36 h after transfection, the cells were fixed with 4% fixative solution (Solarbio) at 4°C overnight, followed by permeabilization with 0.1% Triton X-100 for 30 min at room temperature. The permeabilized cells were blocked with 5% BSA in PBS for 1 h, and then incubated with antibodies (1 μg/mL) for 1 h at room temperature. The cells were washed three times with PBS and incubated with Alexa Fluor 488 Goat Anti-Mouse IgG (H+L) for 1 h at room temperature. After three washes, the cells were incubated with DAPI (4′,6-diamidino-2-phenylindole; Beyotime) for 15 min to stain the nuclei. Images were captured using a LSM880 confocal microscope (Zeiss).

### 2.7 Enzyme-linked immunosorbent assay

The recombinant mAbs were labeled with horseradish peroxidase (HRP) using sodium periodate. Then, each well of the 96-well microtiter plates (Greiner) were coated overnight at 4°C with 100 μL of coating buffer (0.05 M carbonate/bicarbonate buffer, pH 9.6) containing purified CK/SD008 proteins as inactivated whole virus particles at a concentration of 5 μg/mL. After blocking, the coated plates were incubated with serial, two-fold dilutions (1:100 to 1:6400) of HRP-labeled Re-mAbs (the initial concentration was 1 mg/mL) at 37°C for 1 h. After washing three times, 100 μL of tetramethylbenzidine (TMB; Abcam) substrate was added to each well, and the plate was incubated in the dark at 37°C for 10 min. Then, the reaction was terminated with 0.25% HF. The biological activity of the recombinant antibodies was determined at a wavelength of 655 nm.

### 2.8 Surface plasmon resonance

The SPR analysis was conducted on the Biacore 8 K platform (GE Healthcare, Life Science, Milan, Italy) using a Sensor Chip NTA (Cytiva). The HA proteins of CK/SD008 were obtained from the Expi293 Expression System, purified using Ni-NTA, and captured on different channels of an NTA chip using the NTA Reagent Kit (Cytiva) at a density of approximately 300 relative units (RU). CHO-produced recombinant antibodies or mAbs from ascites at 0.03125, 0.0625, 0.125, 0.25, and 0.5 μM were flowed through the channels in six cycles, and the association kinetics were assessed over 180 s at a flow rate of 30 μL/min. Dissociation kinetics were then measured with running buffer at a flow rate of 30 μL/min for a duration of 600 s. The same protocol was used for each binding cycle. The KD of all antibodies with CK/SD008-HA protein was calculated using the Biacore Insight Evaluation Software v3.0.12.

## 3 Results

### 3.1 Generation and characterization of mAbs against CK/SD008-HA

Six hybridoma cell lines (1H9, 2B3, 4F2, 1G4, 1H3, and 4G4) that stably secrete mAbs against CK/SD008-HA protein were screened by ELISA using plates coated with the immunizing antigen and HI assays. Mouse ascites was obtained by injecting hybridomas into paraffin-treated BALB/c mice. Hybridoma supernatants and ascites were collected from the mice for further characterization. The ELISA titers of the six obtained mAbs hybridoma supernatants ranged from 1:10^2^ to 1:10^3^, and the titers of mAbs from ascites were 1:10^4^ to 1:10^8^ (Table 1). The HI titers of the six mAbs from the hybridoma supernatants were 1:2^2^-1:2^3^ and the HI titers from ascites were 1:2^5^-1:2^11^ (Table 1). Isotype identification revealed that all six mAbs had kappa light chains, whereas the heavy chains of 1H9/2B3 and 4F2/1G4/1H3/4G4 belonged to subclasses IgG2a and IgG1, respectively (Table 1). Both the HI titer and ELISA titer of the 1H9 and 2B3 antibodies are higher than those of the other four antibodies. Therefore, these two antibodies were selected for subsequent experiments.

**Table 1:** Characterization of mAbs against CK/SD008-HA.

**mAb**	**ELISA titer of supernatants (log10)**	**ELISA titer of ascites (log10)**	**HI titer of supernatants (log2)**	**HI titer of ascites (log2)**	**Subtype**
1H9	3	8	3	11	IgG2a
2B3	3	6	3	9	IgG2a
4F2	2	4	2	6	IgG1
1G4	3	6	2	6	IgG1
1H3	3	5	2	6	IgG1
4G4	2	4	2	5	IgG1

### 3.2 Molecular characteristics of the mAbs

Two hybridoma cell lines (1H9 and 2B3) were selected for antibody gene sequencing, and the main variable region and partial constant region of the heavy and light chains were amplified using several PCRs. After comparison using Igblast (2023), the V, D, J, and C regions of the 1H9 and 2B3 heavy chains were determined by the germline genes IGHV3-2^*^02/IGHD1-1^*^01/IGHJ3^*^01/IGHG2A^*^01_BALB/c and IGHV3-2^*^02/IGHD2-3^*^01/IGHJ1^*^01/IGHG2A^*^01_BALB/c, respectively, and the light chain V, J, and C regions of the 1H9 and 2B3 antibodies were determined by the germline genes IGKV15-103^*^01/IGKJ2^*^01/IGKC^*^01_musculus (Table 2). The sequencing alignment results are shown in Table 2. Primers were designed based on the germline genes to amplify the constant regions of the light and heavy chains using PCR. Finally, 5′ RACE and 3′ RACE were used to obtain the end fragments of the antibody heavy and light chain cDNAs, and then the complete gene fragments of 1H9H, 1H9L, 2B3H, and 2B3L were amplified.

**Table 2:** Genetic analysis of mAbs against CK/SD008-HA.

	**VH**			**VL**		
	**Length** ^a^	**Mismatches** ^b^	**CDR3** ^c^	**length** ^a^	**Mismatches** ^b^	**CDR3** ^c^
1H9	294	4	ARSDGWYGFAY	284	4	QQCQTYPFT
2B3	293	8	TRDGPYWHIDV	284	7	QQGQSFPYT

^a^Variable genes length of the heavy or light chains. ^b^Mismatched genes of the VH or VL regions between the mAbs and top matched germline antibody. ^c^Complementarity determining Region 3.

### 3.3 Expression and purification of the recombinant antibodies

**Figure 2:**
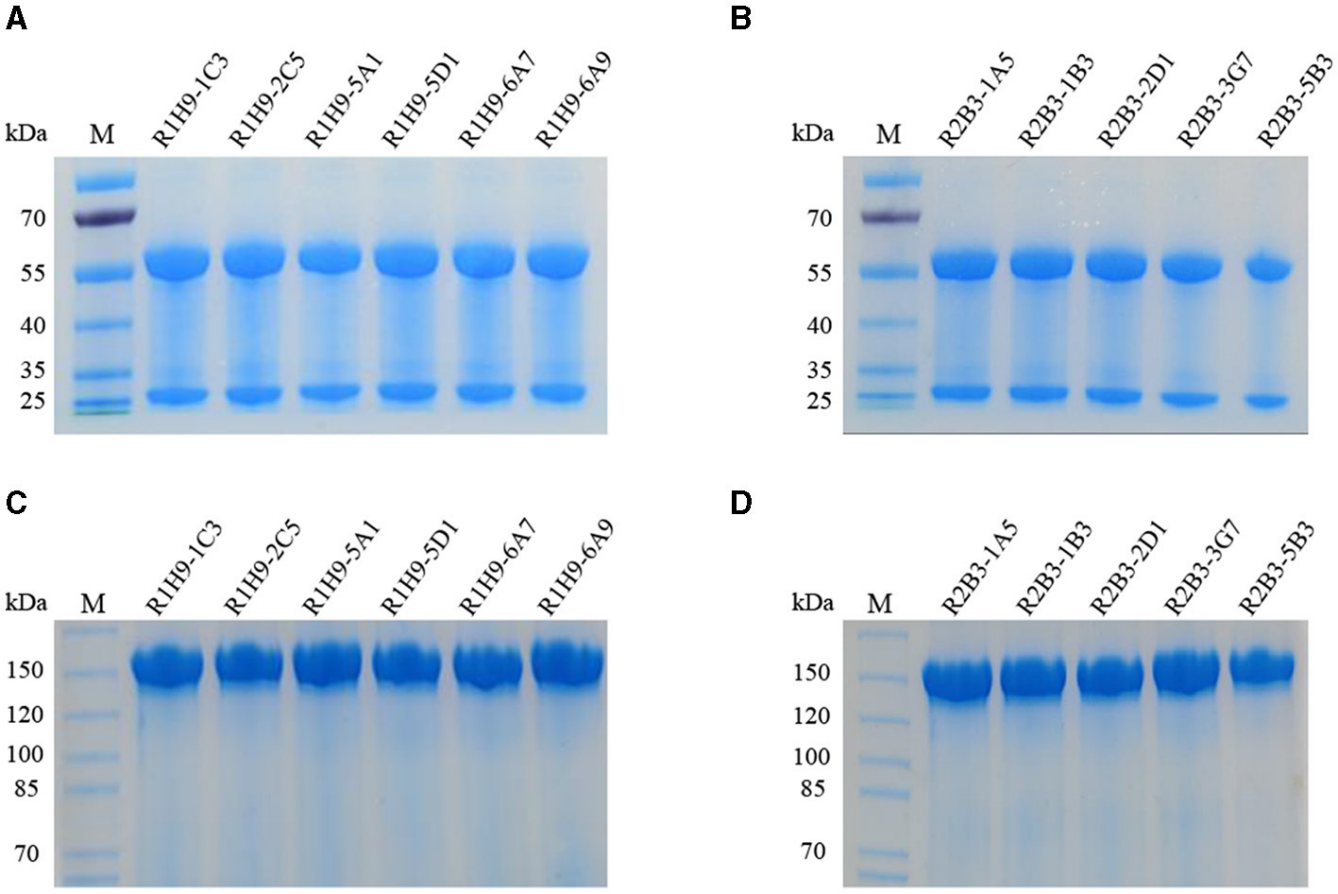
Expression and purification of recombinant antibodies. SDS-PAGE assay with recombinant antibodies R1H9 **(A)** and R2B3 **(B)** to determine their molecular weight. Native-PAGE assay with R1H9 **(C)** and R2B3 **(D)** to determine their molecular weight.

The heavy chain and light chain genes of 1H9 and 2B3 were cloned into the pCHO-1.0 expression vector to construct pCHO-1.0-1H9H-1H9L and pCHO-1.0-2B3H-2B3L, respectively. These two plasmids were electrotransfected into CHO-S cells, and cell lines stably expressing 1H9 and 2B3 antibodies were screened using puromycin and methotrexate. The two cell lines were seeded into 96-well plates. Six cell lines expressing the monoclonal 1H9 antibody (R1H9-1C3, R1H9-2C5, R1H9-5A1, R1H9-5D1, R1H9-6A7, and R1H9-6A9) and five cell lines expressing the monoclonal 2B3 antibody (R2B3-1A5, R2B3-1B3, R2B3-2D1, R2B3-3G7, and R2B3-5B3) were screened using ELISA. Cell suspension culture was performed, and the proteins in the culture supernatant were purified using ProteinG affinity chromatography to obtain the recombinant antibodies. SDS-PAGE and native PAGE showed that the antibodies were effectively purified from the supernatants (Figures 2A–D). These results demonstrated that the recombinant antibody was highly expressed in the cell culture supernatant.

### 3.4 R1H9 and R2B3 can be used in HI assays and for WB analysis

**Figure 3:**
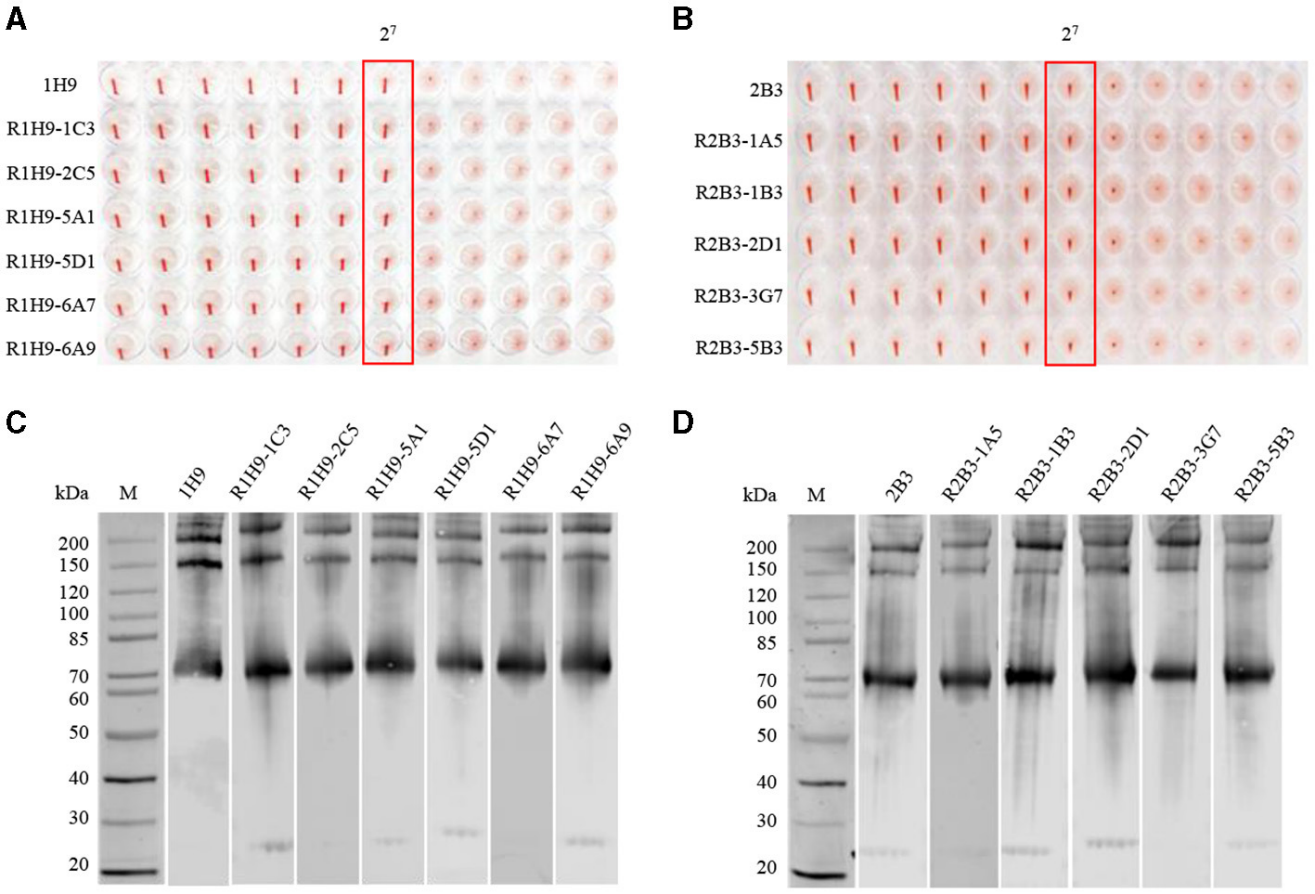
Comparing recombinant and parental antibodies in hemagglutination inhibition (HI) assays and western blot (WB) analysis. The initial concentration of 1H9 and R1H9 **(A)** and 2B3 and R2B3 **(B)** was 0.5 mg/mL. WB to determine the binding of each monoclonal antibody (mAb) to HA protein. Virus lysates were subjected to WB analysis with R1H9 **(C)** and R2B3 **(D)**, respectively.

To evaluate the activity of the recombinant mAbs, purified R1H9 (R1H9-1C3, R1H9-2C5, R1H9-5A1, R1H9-5D1, R1H9-6A7, and R1H9-6A9) and R2B3 (R2B3-1A5, R2B3-1B3, R2B3-2D1, R2B3-3G7, and R2B3-5B3) antibodies were diluted to 0.5 mg/mL for analysis. The HI test results showed that the HI titers of the R1H9 recombinant antibody and the 1H9 antibody obtained from the ascites of BALB/c mice reached 2^7^ (Figure 3A). The six recombinant R2B3 antibodies were also tested using the same procedure. The results showed that the HI titers of the recombinant R2B3 and 2B3 antibodies also reached 2^7^ (Figure 3B). These results showed that there was no significant difference in the HI activity between the recombinant R1H9 and R2B3 antibodies and the respective parent antibodies 1H9 and 2B3. As these antibodies target conformational epitopes of the HA protein, non-denaturing polyacrylamide gel electrophoresis (native-PAGE) was used to separate the CK/SD008 influenza virus lysates. The results showed that the six R1H9 antibodies and five R2B3 antibodies could react with non-denatured CK/SD008-HA, and their binding was similar to that of the 1H9 and 2B3 antibodies (Figures 3C, D). These results confirmed that the recombinant antibody could replace the parent antibody in HI and WB assays.

### 3.5 Application of R1H9 and R2B3 in confocal microscopy

**Figure 4:**
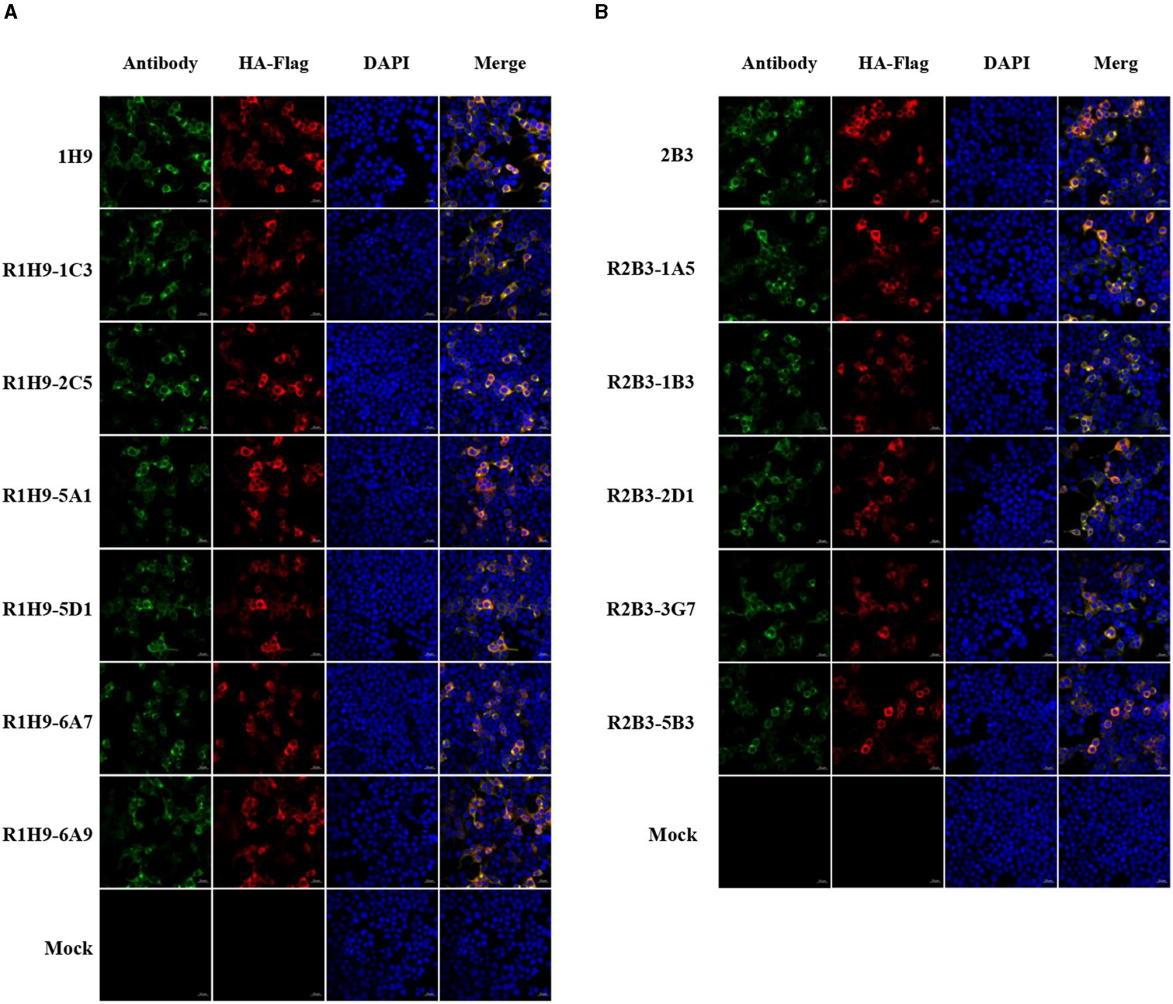
Application of mAbs R1H9 and R2B3 in confocal microscopy. HEK-293T cells were transfected with an HA-expressing plasmid (pCAGGS-SD008HA-flag) or an empty pCAGGS plasmid as a mock/control. At 24 h post-transfection, the cells were subjected to confocal microscopy with either R1H9 **(A)** or R2B3 **(B)** as the primary antibody.

The ability of recombinant mAbs R1H9 and R2B3 to bind to CK/SD008-HA was also assessed by confocal microscopy. HEK-293T cells were transfected with a pCAGGS-CK/SD008-HA-flag plasmid or an empty pCAGGS plasmid as a mock/control. After transfection, the cells were subjected to confocal microscopy using the R1H9, 1H9, R2B3, and 2B3 antibodies. As shown in Figures 4A, B, the R1H9 (R1H9-1C3, R1H9-2C5, R1H9-5A1, R1H9-5D1, R1H9-6A7, and R1H9-6A9) and R2B3 (R2B3-1A5, R2B3-1B3, R2B3-2D1, R2B3-3G7, and R2B3-5B3) recombinant antibodies were able to stain CK/SD008-HA, and the staining effect was similar to that of the parent 1H9 and 2B3 mAbs obtained from ascites, on the premise of ensuring the successful expression of HA protein in HEK-293T cells. These results showed that the recombinant antibodies R1H9 and R2B3 could be used in confocal microscopy instead of the parental antibodies.

### 3.6 Application of the recombinant antibodies for ELISA

**Figure 5:**
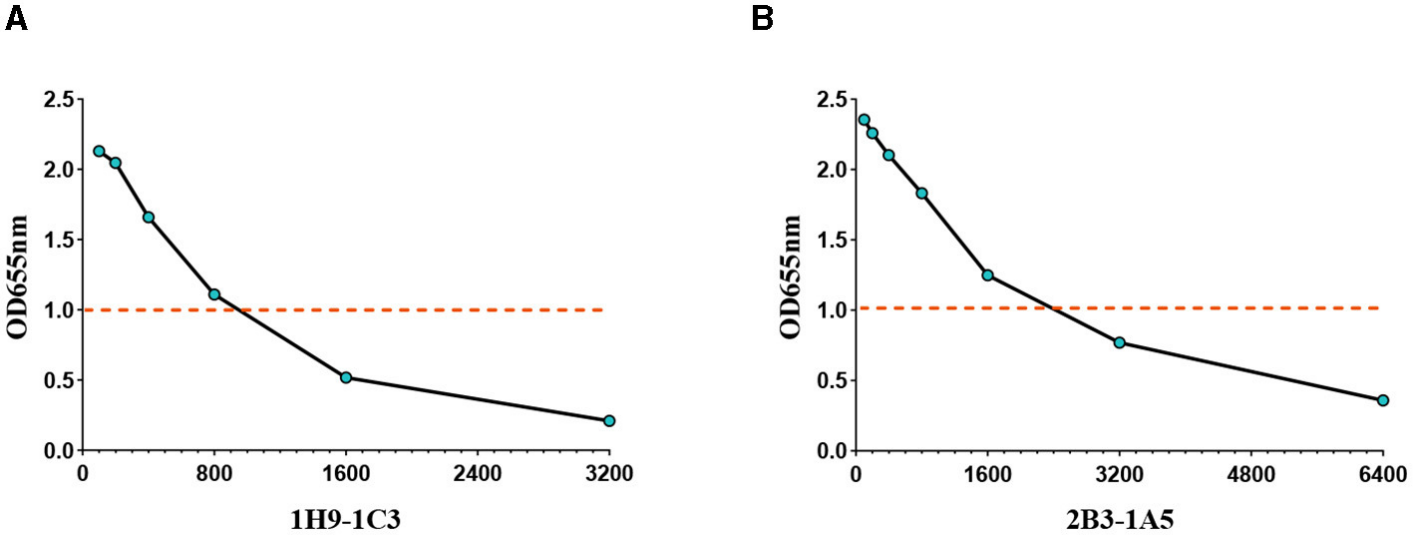
Application of the recombinant antibodies R1H9 and R2B3 in an ELISA to determine their usefulness as enzyme-labeled antibodies. The OD655 of HA with different dilutions of enzyme-labeled HRP-R1H9-1C3 **(A)** and HRP-R2B3-1A5 **(B)** antibodies. The initial concentration was 1 mg/mL.

The activity of the recombinant antibodies R1H9-1C3 and R2B3-1A5 was assessed using ELISA. R1H9-1C3 and R2B3-1A5 were labeled with horseradish peroxidase (HRP) using NaIO4 oxidation. Then, the R1H9-HRP and R2B3-HRP antibodies were two-fold serially diluted (1:100–1:6400) and tested using direct ELISA. The results showed that when HRP-R1H9-1C3 and HRP-R2B3-1A5 were diluted to 1:800 and 1:1600, respectively, the OD655 nm was >1.0, indicating that the recombinant antibody reacted well in the ELISA (Figures 5A, B). These results confirmed that antibodies R1H9 and R2B3 could be used for ELISA.

### 3.7 SPR binding analysis of R1H9 and R2B3

**Figure 6:**
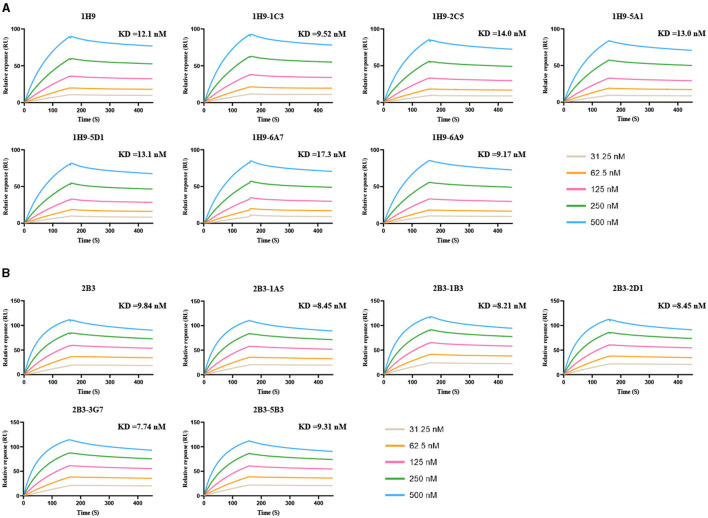
KD values for binding of the mAbs to HA were determined using surface plasmon resonance (SPR). HA was captured in different channels, and 1H9 or R1H9 **(A)** and 2B3 or R2B3 **(B)** were flowed through the channels in six cycles at 31.25 (brown), 62.5 (yellow), 125 (red), 250 (green), and 500 nM (blue).

The KD values for the binding of 1H9, R1H9-1C3, R1H9-2C5, R1H9-5A1, R1H9-5D1, R1H9-6A7, and R1H9-6A9 to SD008 HA were 12.1, 9.52, 14.0, 13.0, 13.1, 17.3, and 9.17 nM (Figure 6A), respectively, showing that all these mAbs had similar affinities for their target antigens. The KD values for the binding of 2B3, R2B3-1A5, R2B3-1B3, R2B3-2D1, R2B3-3G7, and R2B3-5B3 to HA were 9.84, 8.45, 8.21, 8.45, 7.74, and 9.31 nM, respectively, indicating similar affinities for their antigen (Figure 6B). These results showed that both R1H9 and R2B3 had high affinities for HA, and there was no significant difference between these recombinant antibodies and the corresponding parental antibodies purified from ascites.

## 4 Discussion

H7 viruses have caused numerous outbreaks in poultry (Shi et al., 2018), resulting in significant economic losses and public health concerns in recent decades. Effective serological testing in poultry can identify immune individuals and determine whether they have a history of mild or asymptomatic infections, which is crucial for the control and research of H7 AIV (He et al., 2013). Successful serological diagnostic tests depend on high-quality antibodies (Lobert et al., 2013; O'Hurley et al., 2014; Eisinger et al., 2015; Weller, 2016; Andersson et al., 2017).

Since its discovery by Kohler and Milstein (1975), hybridoma technology has become a widely used method for producing monoclonal antibodies. However, a significant drawback of hybridoma-derived antibodies is that genetic drift can lead to variability between batches. In the worst-case scenario, the antibody genes may be completely lost, halting antibody production altogether (Bradbury and Pluckthun, 2015; Bradbury et al., 2018; Parray et al., 2020). Additionally, hybridoma cells can sometimes harbor two or more antibody-encoding genes, resulting in the production of a mixture of mAbs with varying affinities for the target antigen (Duan and Pomerantz, 1994; Ding et al., 2010; Bradbury et al., 2018).

Recombinant antibodies offer significant advantages over conventional antibodies, including high specificity, sensitivity, and stability (Kennedy et al., 2018). Additionally, they can be easily optimized through genetic and chemical modifications (Orlandi et al., 1989; Ward et al., 1989; Kunert and Reinhart, 2016). Furthermore, the production of recombinant antibodies typically requires fewer animals, which enhances animal welfare (Sastry et al., 1989; Dumont et al., 2016; Zhao et al., 2016; Laustsen et al., 2021).

In this study, six murine mAbs were generated against the HA protein of CK/SD008, and their reactivity with H7 subtype AIV was verified by HI and ELISA. The two strains of monoclonal antibodies (1H9 and 2B3) with the highest HI titers were selected and sequenced. Sequence alignment showed that the V, D, J, and C regions of the 1H9 and 2B3 heavy chains were determined by the germline genes IGHV3-2^*^02/IGHD1-1^*^01/IGHJ3^*^01/IGHG2A^*^01_BALB/c and IGHV3-2^*^02/IGHD23^*^01/IGHJ1^*^01/IGHG2A^*^01_BALB/c, respectively, and the light chain V, J, and C regions of the 1H9 and 2B3 antibodies were determined by the germline genes IGKV15103^*^01/IGKJ2^*^01/IGKC^*^01_musculus. The heavy chain and light chain of 1H9 and 2B3 were cloned into the pCHO-1.0 expression vector and transfected into CHO-S cells, and cell lines that stably expressed these antibodies were established. After cell suspension culture, the recombinant expressed antibody, with a molecular weight of approximately 150 kDa, was successfully obtained under native-PAGE conditions.

To evaluate the activity of the recombinant antibodies in serological applications, they were tested in HI assays, western blotting, confocal microscopy, and ELISA. The results showed that the activity of the recombinant antibody was similar to that of the parent antibody. Furthermore, kinetic analysis of the two antibodies against the HA protein of the AIV in the SPR assay showed concordance. The data showed that recombinant mAbs produced using this protocol could replace mAbs obtained from ascites. As recombinant antibody technologies continue to improve, they will likely become the primary method for producing mAbs, and thus recombinant antibodies will play an increasingly important role in the diagnosis of H7 influenza.

## Data availability statement

The original contributions presented in the study are included in the article/supplementary material, further inquiries can be directed to the corresponding author.

## Ethics statement

The animal study was approved by Committee on the Ethics of Animal Experiments of the Harbin Veterinary Research Institute (HVRI) of the Chinese Academy of Agricultural Sciences (CAAS). The study was conducted in accordance with the local legislation and institutional requirements.

## Author contributions

SW: Methodology, Writing – original draft, Writing – review & editing, Data curation, Validation. YZ: Writing – original draft, Writing – review & editing, Methodology. XZ: Writing – original draft, Writing – review & editing. YM: Writing – original draft, Writing – review & editing. JS: Writing – original draft, Writing – review & editing. YJ: Writing – original draft, Writing – review & editing. YL: Writing – original draft, Writing – review & editing. GT: Writing – original draft, Writing – review & editing. XW: Methodology, Resources, Supervision, Writing – original draft, Writing – review & editing.
